# Identifying Postpartum Intervention Approaches to Reduce Cardiometabolic Risk Among American Indian Women With Prior Gestational Diabetes, Oklahoma, 2012–2013

**DOI:** 10.5888/pcd12.140566

**Published:** 2015-04-02

**Authors:** Emily J. Jones, Michael Peercy, J. Cedric Woods, Stephany P. Parker, Teresa Jackson, Sara A. Mata, Shondra McCage, Sue E. Levkoff, Jacinda M. Nicklas, Ellen W. Seely

**Affiliations:** Author Affiliations: Michael Peercy, Teresa Jackson, Shondra McCage, Chickasaw Nation Department of Health, Ada, Oklahoma; J. Cedric Woods, Institute for New England Native American Studies, University of Massachusetts Boston, Massachusetts; Stephany P. Parker, Sara A. Mata, Department of Nutritional Sciences, Oklahoma State University, Stillwater, Oklahoma; Sue E. Levkoff, College of Social Work, University of South Carolina, Columbia, South Carolina; Jacinda M. Nicklas, Division of General Internal Medicine, University of Colorado School of Medicine, Boulder, Colorado; Ellen W. Seely, Division of Endocrinology, Diabetes and Hypertension, Brigham and Women’s Hospital/Harvard Medical School, Boston, Massachusetts.

## Abstract

**Introduction:**

Innovative approaches are needed to reduce cardiometabolic risk among American Indian women with a history of gestational diabetes. We assessed beliefs of Oklahoma American Indian women about preventing type 2 diabetes and cardiovascular disease after having gestational diabetes. We also assessed barriers and facilitators to healthy lifestyle changes postpartum and intervention approaches that facilitate participation in a postpartum lifestyle program.

**Methods:**

In partnership with a tribal health system, we conducted a mixed-method study with American Indian women aged 19 to 45 years who had prior gestational diabetes, using questionnaires, focus groups, and individual interviews. Questionnaires were used to identify women’s cardiometabolic risk perceptions and feasibility and acceptability of Internet or mobile phone technology for delivery of a postpartum lifestyle modification program. Focus groups and individual interviews were conducted to identify key perspectives and preferences related to a potential program.

**Results:**

Participants were 26 women, all of whom completed surveys; 11 women participated in focus group sessions, and 15 participated in individual interviews. Most women believed they would inevitably develop diabetes, cardiovascular disease, or both; however, they were optimistic that they could delay onset with lifestyle change. Most women expressed enthusiasm for a family focused, technology-based intervention that emphasizes the importance of delaying disease onset, provides motivation, and promotes accountability while accommodating women’s competing priorities.

**Conclusions:**

Our findings suggest that an intervention that uses the Internet, text messaging, or both and that emphasizes the benefits of delaying disease onset should be tested as a novel, culturally relevant approach to reducing rates of diabetes and cardiovascular disease in this high-risk population.

## Introduction

American Indians (AIs) experience type 2 diabetes mellitus (DM) and cardiovascular disease (CVD) at twice the rate of the US general population, and cardiometabolic disparities in morbidity and mortality rates are increasing in AIs (1–4). High CVD burden is partially attributable to DM prevalence, which increased from 9.3 to 20.2 per 1,000 population among childbearing AI women younger than 35 years from 1994 to 2004 (5). Women with a history of gestational diabetes mellitus (GDM) have a 20% to 50% chance of developing DM in the 5 to 10 years following GDM (6–8), and cumulative incidence approaches 70% in AI women (9). These women are also more likely to experience CVD events, even in the absence of concurrent DM (8). Diabetes is in itself a major risk factor for CVD, and women with DM who experience a CVD event have worse survival and poorer quality of life than men (10). A post-hoc analysis of self-reported women with prior GDM in the Diabetes Prevention Program demonstrated that women, including AI women, randomized to an intensive lifestyle program had a 53% risk reduction for DM (11).

Because GDM diagnosis may heighten a woman’s risk perception for her health and that of her offspring, the childbearing years present a unique window of opportunity for prevention (12–14). Despite the promise of lifestyle change to decrease cardiometabolic risk, implementing interventions to reduce weight and increase physical activity is challenging because of childbearing women’s busy lives (15,16). Furthermore, AI women with prior GDM may face challenges adopting healthy behaviors because of individual, interpersonal, and sociocultural influences (14). Little is known about AI women’s risk perceptions and prevention beliefs or their perspectives about barriers and facilitators to risk-reducing behaviors postpartum. Given the cardiometabolic risk GDM confers and the potential impact of early intervention, we sought to elicit perspectives of AI women with prior GDM to inform the development of a postpartum lifestyle modification intervention to be tested in this tribal community.

## Methods

We conducted a cross-sectional study of AI women with prior GDM using questionnaires and focus groups and individual interviews from December 2012 to September 2013, in partnership with a large tribal health system in south-central Oklahoma. The institutional review boards of the University of Massachusetts Boston and the Chickasaw Nation of Oklahoma approved this study, and all women provided informed consent.

To recruit participants, providers from the Chickasaw Nation Department of Health (CNDH) and the study team, including staff from the Chickasaw Nation Nutrition Services, which administers the Special Supplemental Nutrition Program for Women, Infants, and Children (WIC), displayed study fliers and distributed business cards with the research team’s contact information in the medical center and community clinics. Eligibility criteria were the following: 1) self-identified as AI, 2) aged 19 to 45 years, 3) health care obtained through CNDH, and 4) diagnosed with GDM within past 10 years (confirmed by chart review) but not currently in a first-GDM pregnancy. Of 49 women who expressed interest in participating and were considered eligible, we scheduled 28 for participation. Of those who expressed interest but did not participate, many were either unable to schedule a focus group or interview or did not return follow-up telephone calls or emails. Five of the 28 participants had been diagnosed with DM since their GDM pregnancy; these women did not complete the Risk Perception Survey for Developing Diabetes (RPS-DD) but their data were included in all other analyses.

### Data collection procedures

We offered focus groups at various times of the day and week over a period of months, and only 12 of 28 women interested in participating in focus groups attended. During one focus group, we learned a participant was in her first GDM pregnancy and not eligible for the study; therefore, we present focus group data from 11 women. We invited the other 16 women to complete face-to-face, individual interviews; 13 women completed these, and several of these interviews were rescheduled to accommodate the women. Two women completed surveys or interviews by telephone, because they lived far from the tribal headquarters. One woman did not participate in a focus group or interview due to an unanticipated surgery that complicated her schedule in the study time frame. Therefore, for these analyses, we present data from a final sample of 26 women.

We (S.P.P., T.J.) conducted 4 comoderated focus groups consisting of 2 to 5 participants and lasting approximately 60 minutes. Interviews ranged from 25 to 45 minutes and were conducted by 2 team members (T.J., S.M.). Participants completed surveys before focus groups and interviews. All focus groups and interviews were audio-recorded and transcribed. Participants received a $20 gift card for focus group and interview participation and a $10 gift card for survey completion.

### Measures

Participants completed a demographic questionnaire on personal and family health history and technology (Internet, mobile telephone) feasibility and acceptability. To measure risk perception for DM, we used 2 risk perception questions from the RPS-DD (17). We adapted these questions to measure risk perception for CVD (termed “heart disease”); only women who already had DM answered the CVD-related questions.

The focus group/interview moderator’s guide included questions about women’s risk perceptions and health beliefs regarding DM and CVD prevention, barriers and facilitators to healthy lifestyle behaviors postpartum, and modes of participant engagement to inform a lifestyle modification intervention. We asked women to reflect on their postpartum experiences following GDM, including barriers and facilitators to lifestyle change, and to describe characteristics of a program that might have helped them to carry out risk-reducing behaviors. Researchers with extensive familiarity in the subject matter and experience conducting qualitative research in this community reviewed the guide for face validity.

### Data analysis

We analyzed and interpreted focus group and interview data by using inductive content analysis to identify codes, subcategories, categories, and overarching themes (18). Two team members (E.J.J., E.W.S.) independently examined the transcripts and manually divided text into meaning units reflecting words or sentences containing aspects related to each other through their content and context. We condensed meaning units into codes, sorted these into categories and subcategories, and interpreted the underlying meaning of the categories into themes. We conducted a subset analysis of women with DM to identify additional themes. The 2 coders met to compare codes, resolve discrepancies, and validate the coding scheme. A third team member (S.E.L.) met with the 2 coders to review coding and categorization and reach consensus on representative data and a final set of themes. We selected quotes to illustrate major themes. For survey items, we calculated frequencies, means, and standard deviations (SDs).

## Results

### Demographics

The mean age of the 26 participants was 32 (SD, 4.8) years, and participants had a mean number of 2.3 (SD, 0.7) children. The average length of time since most recent GDM was 3.7 (SD, 3.1) years (1 woman whose data were included in analyses was in her second GDM pregnancy at time of focus group). DM had been diagnosed in 5 of 26 women in the previous month to 5 years, and 11 of 26 reported a history of depression. All women reported a family history of DM, and most reported hypertension or CVD in a first-degree family member (Table 1).

**Table 1:** Characteristics of Participants in an Exploratory Study to Identify Postpartum Intervention Approaches to Reduce Cardiometabolic Risk in American Indian Women With Prior Gestational Diabetes, Oklahoma, 2012–2013^a^

Characteristic	Overall Sample (N = 26)
**Age, y, mean (SD)**	32 (4.8)
**Number of children, mean (SD)**	2.3 (0.7)
**Length of time since GDM, y, mean (SD)**	3.7 (3.1)
**Marital status**	
Single	2
Married or living with partner	23
Divorced	1
**Education**	
Some high school (9th through 11th grade)	1
High school graduate or GED	9
Some college or vocational training	6
Associate degree	3
Bachelor’s degree or higher	7
**Employment^b^**	
Currently employed	15
Out of work and looking for work	1
Homemaker	5
Student	1
Unable to work	1
**Self-reported personal health history^c^**	
Type 2 diabetes	5
Depression	11
Smoked at least 1 cigarette in previous 18 months	12
**Self-reported family history of disease (in a first-degree family member)^c^**	
Diabetes mellitus	26
Heart disease	22
Hypertension	22
Stroke	16
**Daily Internet access**	
Always	17
Most of the time	7
Some of the time	2
**Own mobile phone with text messaging plan**	26
**Frequency of sending and receiving text messages**	
Daily	25
Weekly	1

Abbreviation: SD, standard deviation; GED, general educational development certificate; GDM, gestational diabetes mellitus.

a Data presented are whole numbers unless otherwise indicated.

b n = 23 due to missing data.

c Respondents could choose more than 1 answer.

Most participants (24 of 26) reported accessing the Internet daily from home or another convenient location “always” or “most of the time”; only 2 of 26 reported occasional poor connectivity. All participants reported access to a mobile telephone with texting plan; 25 of 26 reported sending and receiving text messages daily, and 24 of 26 reported having an unlimited plan. Nineteen of 26 reported engaging in social networking daily; only 2 women reported never engaging with social networking. There were no significant differences in demographic characteristics between women who participated in focus groups and women who were interviewed.

### Risk perception

Among participants without DM, 15 of 19 reported a moderate to high chance of developing DM in the next 10 years, and the number increased (17 of 19) when asked to assess risk in the absence of lifestyle behavioral change. Nearly half of all participants (12 of 26) reported having a moderate to high chance of developing CVD in the next 10 years, and 19 of 26 reported a moderate to high chance without lifestyle changes. Among the subset diagnosed with DM, 4 of 5 reported having a moderate to high chance of developing CVD in the next 10 years with no lifestyle changes; 1 woman with DM perceived her risk as slight (Table 2).

**Table 2:** Risk Perception for Developing Type 2 Diabetes and Heart Disease Among American Indian Women With Prior Gestational Diabetes, Oklahoma, 2012–2013

Risk Perception	No.^a^
**For developing diabetes over the next 10 years^b^ (n = 19)**	
Almost no chance	1
Slight	3
Moderate	12
High	3
**For developing diabetes over the next 10 years without changing lifestyle behaviors^b^ (n = 19)**	
Almost no chance	0
Slight	2
Moderate	9
High	8
**For developing heart disease over the next 10 years^c^ (n = 26)**	
Almost no chance	5
Slight	9
Moderate	9
High	3
**For developing heart disease over the next 10 years without changing lifestyle behaviors^c^ (n = 26)**	
Almost no chance	1
Slight	6
Moderate	9
High	10

a Data presented are whole numbers unless otherwise indicated.

b n = 19 due to 2 cases of missing data among participants without diagnosed type 2 diabetes (n = 21).

c Heart disease risk perception questions were administered to all participants (n = 26).

### Qualitative findings

Major themes and representative quotes related to women’s prevention beliefs, perceived barriers and facilitators to postpartum lifestyle modification, and perspectives about a potential lifestyle modification program were consistent across focus groups and interviews and spanned individual, relational, community, and sociocultural domains (Table 3).

**Table 3:** Qualitative Themes Identified During Focus Groups and Individual Interviews Discussing Prevention Beliefs and a Potential Lifestyle Modification Program for American Indian Women With Prior Gestational Diabetes, Oklahoma, 2012–2013

Theme	Representative Quote
**Domain 1: Beliefs About Prevention and Delay of DM and CVD**	
High risk perception for DM and CVD	“I could do a lot of things to help delay [DM], maybe prevent it. Maybe I’m wrong and I just need to work harder . . . but I think it’s one of those things that we’re just genetically unlucky.” (FG4) [Regarding heart disease] “I think no matter how much you make yourself toe the line, eating right and everything else, inadvertently genetics is always gonna come and kind of pull out the ace and trump you.” (II6)
Delay of DM and CVD more likely than prevention	“I don’t think it’s so much as preventing [DM] as much but there is delaying it. You can delay the process . . . like it’s gonna take its time, but if you slow the process down it will take that much longer before you actually do [develop DM].” (FG1)
Inevitability of DM related to family history	“I think I will have diabetes in the future because all of my family does.” (FG3)
Importance of attempting to change lifestyle behaviors to minimize severity of future disease	“If you just say I’m destined or doomed for [heart disease] just because you’re genetically predisposed, you’re in essence cutting your life short altogether. . . . To delay it you’re gonna have longevity. . . . I think it’s really important that you attempt to prevent it.” (II1)
Knowledge-behavior gap	“I know what the right choices are, I just choose not to make them sometimes . . . but I’m making an informed decision.” (FG4)
Perceived lack of knowledge about heart disease	“I don’t know much about heart disease or anything like that, but it’s a muscle and if you don’t work your muscle, it’s not gonna work for you, so diet and exercise should help prevent a lot of things.” (FG4)
**Domain 1a: DM and CVD Beliefs — Perspectives of Women Who Had Developed DM**	
Attempting to minimize DM severity and reduce heart disease risk	“Now that I have diabetes I’m walking . . . so I don’t gain more weight and get any heart disease or heart problems.” (II14)
**Domain 2: Facilitators of and Barriers to Postpartum Lifestyle Change**	
Facilitators Support of family and friends Breaking the cycle of poor health in the family Motivated by fear of developing DM and/or CVD	“My husband is great . . . he’s got high blood pressure, so he has to eat healthier also. So we’re the team with it . . . we support each other.” (II15) “If I’m eating right, then they see me eating right . . . they’re getting those skills and getting the idea it’s important to exercise, to eat right. So hopefully to kind of break the cycle in a way.” (II6) “I’m going to regret it if I don’t change certain things . . . I’m scared that I’m gonna have to go through that [managing DM], so I’m gonna do whatever I can to try and change it.” (II15)
Barriers Competing priorities and demands Social sabotage or a perceived lack of support	“Once you have your baby it’s all about caring for them and what they need, not yourself . . . I don’t have any memory of ‘Did I eat right or did I exercise?’ . . . any of that.” (FG3) “They’ll tease you about how you can’t eat this food, and they put it in front of you . . . try to get you to eat it, and most people go ahead and eat that unhealthy food.” (II10) “[N]ot to have the support in your home can get to you . . . that person wants to do better for themselves.” (II1)
**Domain 3: Benefits of and Barriers to Participating in a Postpartum Intervention Program**	
Benefits Focus on health of entire family Being a role model for children Foster continuation of healthy diet following GDM Increase social support between women in family and community	“If you’re looking at prevention of diabetes, teaching kids at a very young age to eat healthy and to exercise [is key] . . . so [you need] to draw the kids in.” (II1) “We play ball, we ride bikes together . . . so I do feel like I’ve taken some steps [as a role model] . . . but could still do a better job with them [her children] right now.” (II1) “I was just going to my 6-week checkup because the diabetes clinic really didn’t need me anymore because I didn’t need them [after management of GDM pregnancy]. So yeah, a program after I delivered would have been awesome.” (FG3) “It would help other women to see that someone else can do it [be successful with healthy lifestyle changes] and would also be able to help someone that needs that help . . . because, you know, ‘I care and I know how you are and I can help you do it.’” (II13)
Barriers Lack of time and childcare Time away from being with their families Rural residences made traveling to the tribal health headquarters inconvenient and expensive	“I’d rather spend time with my kids and just hang out with them and run around the backyard, as opposed to taking time away from them, so . . . it’s kind of like a tug, a moral tug in a way, so I put my stuff on the back burner.” (II6) “Obviously it would not be very good to have to drive all the way into town, sit down, talk to someone for 30 to 45 minutes, and then have to drive all the way back in less than an hour. . . . I think location and scheduling would probably be the two biggest challenges.” (II2)
**Domain 4: Preferences for Program Design**	
Increase social support while promoting accountability Provide motivation by connecting with someone relatable Limit face-to-face time to accommodate competing family and work demands Provide smooth transition from pregnancy to postpartum Promote family participation and women’s central role as primary stewards of family health	“We could text motivational things like, ‘so-and-so reached her goal,’ and then we could be happy for her, and that’s gonna make her feel good because we’re all in that group together.” (FG4) “[A] relatable person [in the program] . . . they probably cave too . . . they’re not picture perfect . . . they’re trying as well and they believe that you can do it, and they’re very invested in you as much as you’re invested in a program.” (FG2) “[S]ay it’s during the day, to set up the time from work, to get there, to get back, you have to factor that in. How long are you gonna be there? What if it runs over after work? . . . What to cook for dinner? And things like that.” (II4) “I think initiating this even while they’re still in the hospital. From giving birth, saying, ‘Hey, this is an option out there,’ and starting it right off the bat. And keeping in mind there’s ways to modify it, ’cause you need more calories and things when you’re breastfeeding.” (FG4) “There’s a sense of being the matriarch. You’re a provider, whether it’s financially [or as] a homemaker . . . so [women need] to have guidelines to help change their lifestyle as well as trickle down to their family.” (II11)

Abbreviation: CVD, cardiovascular disease; DM, type 2 diabetes mellitus; GDM, gestational diabetes mellitus; FG, focus group; II, individual interview.

#### Beliefs about prevention and delay

Most participants expressed high risk perception for DM and believed they were more likely to delay DM onset than prevent it altogether. They frequently attributed this belief (delay vs prevention) to their strong family histories. Discussion related to genetics, a term not appearing in the moderator’s guide, surfaced frequently, and it was usually expressed synonymously with family history. Although most participants expressed doubt that preventing DM was possible, most women highlighted many benefits to delaying disease, emphasizing longevity, being healthier and more active in their children’s lives, and controlling the severity of future DM. Several women believed DM prevention was possible for their children and that being a role model was important.

Most participants also expressed high risk perception for CVD and believed that delay was more likely than prevention. The belief that CVD was inevitable, related to family history, was common. The few women who did not have a first-degree family member with CVD expressed that they were more likely to prevent CVD than DM, and several women stated that delay is possible with lifestyle changes, even with a family history. Many women expressed uncertainty and a lack of knowledge about CVD prevention compared with DM, and one woman referred to it as a “silent killer,” more elusive than DM. Several women stated that they presumed that the risk factors were similar for both diseases and that lifestyle behaviors that would delay one would delay the other.

When asked how they could prevent DM and CVD, many women mentioned the importance of consuming a diet high in whole grains, proteins, and fresh fruits and vegetables; being physically active; and not smoking. Several women noted that information related to healthy lifestyle behaviors was easily accessible in this tribal community.

Women with prior GDM who had been subsequently diagnosed with DM attributed this to having a strong family history, being overweight, and not reducing weight after GDM. The women with DM reported currently attempting to eat healthfully and exercise to minimize severity, improve self-management, avoid insulin, and reduce risk for CVD.

#### Perceived benefits and barriers of participating in a postpartum intervention program

All women who participated in focus groups or interviews stated that they would have been, or would be, interested in participating in a risk-reducing lifestyle program for women with prior GDM. Perceived barriers and facilitators to postpartum lifestyle change affected women’s preferences for program design. Common barriers to preventive behaviors included competing priorities, exhaustion, childcare duties, and time-related, financial, and geographic constraints. Across focus groups and interviews, many women expressed a lack of social support as a major barrier to eating healthy postpartum, and in interviews, 3 women expressed experiencing a sense of sabotage from family members when they attempted to eat healthier at home. Facilitators of lifestyle change included the perceived value of role modeling healthy behaviors in the family, social support, nutritional education, and access to gyms with childcare. We also identified common themes related to perceived benefits and barriers of a potential lifestyle modification program (Table 3).

#### Preferences for program design

Most women expressed that the ideal lifestyle program would provide motivation and promote accountability while accommodating women’s competing family and work demands. Many women felt that a mode of program delivery that could maintain a sense of social support and promote family participation while not requiring face-to-face time would best facilitate involvement. Several women also mentioned it would be helpful to initiate such a program during the GDM pregnancy or immediately postpartum to promote smoother transitions and to help women think ahead toward prevention. Several women stated they would be interested in a program that would help them better organize their daily routines to prioritize healthy meal planning and exercise postpartum. In 1 focus group and 1 interview, women mentioned that a program might address potential postpartum depression and increase women’s confidence to carry out healthy lifestyle behaviors.

When asked to consider potential program designs (eg, face-to-face, Web-based, and text-messaging) in an individual or group setting, the most common preference was for a program that primarily used text-messaging with the potential for supplemental sessions with a lifestyle coach, either online or face-to-face. However, in general, women did not feel they would be able to participate face-to-face unless the program was combined with another scheduled appointment for the mother or the baby. Many women mentioned that the timing of text messages would be critical so they would not be disregarded, and they thought that text messages could be educational and motivational and could promote accountability. Women indicated interest in receiving reminders or tips through messaging, even group texts.

## Discussion

To our knowledge, this is the first study to elicit perspectives of AI women with prior GDM to inform the development of a postpartum lifestyle program to reduce cardiometabolic risk to be implemented in a tribal community. We found that most Oklahoma AI women believed they would inevitably develop DM, CVD, or both. Although they were optimistic that they could delay onset or decrease severity of disease, women discussed many individual, relational, and social barriers to postpartum lifestyle change. Most women expressed enthusiasm for a lifestyle change program after pregnancy, incorporating facilitators and using the Internet and text messaging, thereby reducing the need for face-to-face contact, a major barrier to participation.

In our study, Oklahoma AI women expressed moderate to high risk perception for DM and CVD following GDM. In contrast to findings from a study that examined DM risk perception among a sample of predominantly white women (17), a larger proportion of women in our study correctly considered themselves at moderate or high risk for DM. This finding is probably due to women’s pervasive family histories but also may have been, in part, because of the GDM education received through the tribal health system. We also found that AI women with a family history of CVD considered themselves at moderate or high risk for CVD, although many women generally perceived a lack of knowledge and familiarity with CVD compared with DM. This finding should be considered when designing programs for AI women with prior GDM, because these women are at increased risk for CVD (8).

Congruent with findings from an earlier study in this tribal health care system (14), most women in this study believed that the development of DM or CVD was inevitable. When asked in greater depth about their beliefs, women were optimistic that they could delay onset or decrease severity of disease with lifestyle changes. This is a crucial finding that should inform future lifestyle modification interventions in this community. It is possible that the public health message of diabetes prevention is not compatible with women’s beliefs and lived experiences, and messages related to delay may be more effective for certain populations than for others. Further research is needed to assess the value of such tailored messaging.

Although their risk perception was high, women in our study reported similar barriers to adopting lifestyle changes as women with prior GDM in other studies (19,20). Building on the lifestyle changes many women adopt during GDM pregnancies, the best strategy to reduce DM may be to implement tailored, relevant, postpartum lifestyle modification interventions (13). Adding to our 2010 study findings (14) and similar to the findings of a recent study in Montreal among women primarily of European origin (20), we found that women had a high perceived need for social support, particularly from partners and family members, and they expressed enthusiasm for a postpartum lifestyle change program incorporating facilitators to promote health behaviors. However, in contrast to the Montreal study’s finding that in-person interactions with peers and professionals were deemed essential, many Oklahoma AI women felt this approach would be too challenging and potentially not feasible for them, unless it was with an existing appointment. Notably, most participants could access the Internet daily from home and reported engaging daily in social networking. All women had a mobile telephone, and most reported having an unlimited texting plan; all but 1 participant reported sending and receiving text messages daily, a finding similar to trends found in a recent survey of this age group (21). This finding is important given the rural location of participants and other time-constraint–related challenges inherent in a face-to-face program; although women thought such a program would be motivating, they stated numerous barriers to attending it. Similar to findings of recent studies (13,20), women in our study were challenged to attend a single focus group, even when offered numerous options at various times of the day and week over a period of months and when several interviews were rescheduled to accommodate them. Additionally, many women who expressed interest in participating did not return telephone calls or emails from research team members or were unable to schedule an in-person meeting. This finding reflects a critical challenge in translating prevention strategies, as interventions must account for women’s competing priorities and time constraints (15).

One important finding, which should prompt further study, was the overall high rate of self-reported depression in this group of AI women with prior GDM. Tailoring postpartum lifestyle interventions to address depression could be critical to promoting healthy lifestyle change and reducing cardiometabolic risk among women who live with depression.

This study builds on our previous work in that it provides AI women’s perspectives about a postpartum lifestyle change program that would be feasible and acceptable in this community. Our findings suggest that a program using text messaging or the Internet that is tailored to address family based changes and that encourages family involvement may be effective for promoting lifestyle change in women with prior GDM in this tribal community. A recent systematic review described 9 lifestyle intervention studies to reduce DM risk in women with prior GDM, and many of these used a combination of in-person and technology-based modes of delivery; although most were pilot or feasibility studies, preliminary findings suggest lifestyle interventions can reduce DM risk (16). These interventions should be tested in larger, well-designed, randomized controlled trials and tailored to be culturally relevant for high-risk populations.

Our study has limitations. The regional, purposive sample of Oklahoma AI women may limit the generalizability of our findings. Furthermore, our final participation rate (26 of 48 eligible women, or 54%) limits findings, because the 22 women who did not participate may have held different views than participants. In addition to potential self-selection bias, recall and social desirability biases may have affected responses.

A family friendly mobile health or technology-based program that provides motivation and promotes accountability for lifestyle behavioral change, while accommodating women’s competing family and work demands, should be tested to reduce rates of DM and CVD in this high-risk group of AI women with prior GDM. Programs that will effectively delay or prevent DM and CVD in this population require an understanding of the greater sociocultural context, with culturally and situationally relevant tailoring of interventions (22–24). For this group of AI women who largely perceived the development of DM and CVD as inevitable, a tailored approach emphasizing the benefits of delaying disease onset, rather than preventing disease, may be the best approach. Because AI women are usually the primary stewards of family health (25), translating DM and CVD delay or prevention in this group is critical to prevention across the life course, the goal being to eliminate cardiometabolic health disparities in AI communities.
